# Peer Review of “Why We Are Losing the War Against COVID-19 on the Data Front and How to Reverse the Situation”

**DOI:** 10.2196/28720

**Published:** 2021-05-05

**Authors:** Rebecca Krukowski

**Affiliations:** 1 Department of Preventive Medicine The University of Tennessee Health Science Center Memphis, TN United States

**Keywords:** COVID-19, learning health systems

*This is a peer-review report submitted for the paper “Why We Are Losing the War Against COVID-19 on the Data Front and How to Reverse the Situation”.*

## Round 1 Review

### General Comments

This paper [1] is interesting in its examination of the challenges with data use during the COVID-19 pandemic and the recommendations for a system of data collection and management. The authors appropriately address the potential privacy concerns. It is a well-written piece.

### Specific Comments

#### Major Comments

1. The authors should ensure that their points are supported by citations, appropriate to a scientific manuscript.

2. The authors should address several main barriers to the recommendations made—*who* would regulate the sharing of the data across systems and international borders (with potential language differences), *who* would coordinate the analyses conducted with shared data to make sure that there is efficiency (to ensure multiple teams are not conducting the same analyses) and that appropriate analyses are conducted, and *where* would the funding coming from (when most health systems are losing money due to cancellation of nonessential procedures).

3. Not all authors use preprints, and peer review has not been effective in addressing some rushed science, particularly in the last few months. How should these concerns be addressed?

## Round 2 Review

### General Comments

The authors have been responsive to the reviewers’ comments and have improved the piece. I have a few remaining concerns, as noted below.

### Specific Comments

1. The paper could still benefit from references to support the authors’ points. For example, the first paragraph does not include a single reference. As another example, point 7—what evidence do the authors have that, “the more relevant the data of the registry and the larger the potential research community, the bigger the bottleneck is likely to be”?

2. Epidemiological data are, in fact, telling us who is more likely to be infected—by race, ethnicity, age, etc. The authors should clarify the unique contribution of clinical data.

3. It is not clear what the authors mean by “In large health systems that cover most of the population cases can be captured rather quickly if the learning system is implemented and coordinated across sites (hence the need for regulating and standardising the methods).”

4. The authors do not clearly define the health learning system in contrast to current practices.
